# Decoding the Mechanism of the Loss of Self in Spouse Caregivers of Alzheimer’s Patients: A Sociocultural and Communication Perspective

**DOI:** 10.1002/alz.093488

**Published:** 2025-01-09

**Authors:** Erting Sa

**Affiliations:** ^1^ University at Albany, SUNY, Albany, NY USA

## Abstract

**Background:**

The experience of spouse caregivers of individuals with Alzheimer’s Disease (AD) is marked by witnessing the gradual cognitive decline of their loved ones. This journey transforms the nature of their marital relationship, evolving from mutual interdependence to a more unilateral caregiving role. Despite this significant shift, the specific phenomenon of self‐loss among these caregivers remains underexplored in academic research.

**Method:**

Adopting the symbolic interactionist perspectives, which argue the self is a symbolic and relational process, created and sustained through ongoing social interactions, this research conducts an extensive review of interdisciplinary literature, integrating influential theoretical models, including George Mead’s (1967) interacting self, Peter Berger and Hansfried Kellner’s (1964) theories on marriage and reality construction, Wegner, Giuliano, & Hertel’s (1985) work on cognitive interdependence in close relationships, and Timothy Stephen’s (1986) symbolic interdependence theory. These frameworks collectively inform a new theoretical concept to elucidate the nature of self‐loss experienced by AD spouse caregivers.

**Result:**

The proposed theoretical perspective suggests that AD significantly transforms the intimate reality previously co‐constructed by marital partners. The deterioration of the AD‐affected spouse’s language and memory capacities erodes the symbolic coherence that once underpinned the marital relationship. This leads to a profound disruption in the couple’s shared reality, which is essential for affirming and supporting each partner’s self‐identity, beliefs, and values. As a consequence, spouse caregivers not only witness the gradual erosion of their partner’s self but also experience a profound disruption in their own sense of self, particularly in aspects deeply intertwined with their marital identity.

**Conclusion:**

This theoretical exploration offers significant implications at societal, community, interpersonal, and intrapersonal levels. By enriching existing literature, it aids policymakers, researchers, healthcare organizations, and families affected by AD in comprehending the interpersonal impact of the disease on spouse caregivers. Furthermore, this perspective sheds light on the emotional, mental, and psychological challenges faced by caregivers, underscoring the critical need for robust social support systems. Consequently, this research could inform the development of targeted intervention programs and health policies, thereby supporting spouse caregivers who play a crucial role in the healthcare system and in the lives of their loved ones.

